# Analysis of neurexin-neuroligin complexes supports an isoform-specific role for beta-neurexin-1 dysfunction in a mouse model of autism

**DOI:** 10.1186/s13041-025-01183-0

**Published:** 2025-03-14

**Authors:** Francisco Arias-Aragón, Estefanía Robles-Lanuza, Ángela Sánchez-Gómez, Amalia Martinez-Mir, Francisco G. Scholl

**Affiliations:** 1 Instituto de Biomedicina de Sevilla (IBiS), Hospital Universitario Virgen del Rocío/CSIC/Universidad de Sevilla, Seville, Spain; 2 Departamento de Fisiología Médica y Biofísica, Universidad de Sevilla, Seville, Spain

**Keywords:** Neurexin, Neuroligin, Protein complexes, Autism, Synapse

## Abstract

Neurexins are presynaptic plasma membrane proteins that regulate key aspects of synapse physiology through the formation of transcellular complexes with postsynaptic ligands, including neuroligins (Nlgns). Each neurexin gene (*NRXN1*-*3*) generates two main alternative-spliced transcripts that generate alpha and beta-Nrxn isoforms differing in their extracellular domains. Mutations in *NRXN1* are associated with autism and other neurodevelopmental disorders. However, whether dysfunction of *NRXN1* occurs through common or isoform-specific postsynaptic partners for alpha- and beta-Nrxn1 is not completely known. The association of Nrxn1 proteins with postsynaptic partners has been mostly analysed in experiments that test binding, but Nrxn proteins must interact with Nlgns in opposing cells, which requires transcellular oligomerization. Here, we studied the interactions of Nrxn1/Nlgn pairs across the synapse and identified the type of association affected in a mouse model of autism. We found that beta-Nrxn1 can be recruited at synaptic contacts by glutamatergic Nlgn1 and GABAergic Nlgn2, whereas alpha-Nrxn1 is a presynaptic partner of Nlgn2. Insertion of alternative spliced segment 4 (AS4) negatively modulates the presynaptic recruitment of Nrxn1 by Nlgns. These data obtained in transcellular assays help clarify previous knowledge based on the ability of Nrxn1 to bind to Nlgns. Interestingly, we found that a mutant beta-Nrxn1 shows ligand restriction for glutamatergic Nlgn1 in the brain of a mouse model of autism. These findings suggest that autism-associated mutations affecting beta-Nrxn1 can act through specific synaptic partners that may be different from those of its alpha-Nrxn1 counterparts.

**Supplementary Information:**

The online version contains supplementary material available at 10.1186/s13041-025-01183-0.

## Introduction

Neurexins (Nrxns) are presynaptic plasma membrane proteins associated with autism and other neurodevelopmental disorders [1, 2, 3]. Nrxns are encoded by three genes (*NRXN1*, *2* and *3*) that generate thousands of isoforms differing in their extracellular domains via promoter usage and alternative splicing [4, 5, 6, 7]. Each *NRXN* gene generates two main transcripts, long alpha-Nrxns from a 5’ promoter and short beta-Nrxns from an internal promoter [8, 9]. In addition, Nrxn1 expresses a truncated isoform transcribed from the gamma promoter [10]. The extracellular domain of alpha-Nrxn proteins is formed by three EGF (Epidermal Growth Factor)-like domains flanked by two LNS (Laminin, Neurexin, Sex-hormone binding globulin) domains. Following a short specific sequence at the N-terminus, the sequence of beta-Nrxns is identical to that of their alpha-Nrxn counterparts starting at the last LNS domain (LNS6). Alternative splicing greatly increases the molecular diversity of Nrxns. Thus, the extracellular domain of alpha-Nrxn genes contains six alternative spliced segments (AS1-6), two of which (AS4 and 5) are shared by beta-Nrxn genes [4–7]. Human genetic approaches have led to the identification of deletions and point mutations in *NRXN1* in patients with autism and other neurodevelopmental disorders. Autism-associated mutations in *NRXN*1 can affect 5’ isoform-specific sequences in alpha- and beta-Nrxn1 or the 3’ region shared by alpha and beta Nrxn1 isoforms [11–18]. Large deletions affecting both isoform-specific and common *NRXN1* sequences have also been described. Therefore, the identification of autism-associated mutations in *NRXN1* might point to the disruption of a common function shared by alpha- and beta-Nrxn1 isoforms or, rather, the alteration of unique roles in an isoform-dependent manner.

Nrxns act as presynaptic partners for a number of postsynaptic proteins during synaptic differentiation and function. The best-studied postsynaptic ligands for Nrxns are neuroligins (Nlgns). Nlgns form a family of postsynaptic plasma membrane proteins encoded by five genes (*NLGN1*, *2*, *3*, *4X*, and *4Y*), which show differential expression at synapses. Nlgn1 is expressed at glutamatergic synapses, Nlgn2 at GABAergic synapses, and Nlgn3 can be located at glutamatergic and GABAergic synapses [19, 20, 21]. The formation of signalling complexes containing presynaptic Nrxns and postsynaptic Nlgns is thought to regulate the function of glutamatergic and GABAergic synapses. Therefore, disruption of Nrxn/Nlgn signalling might alter the excitation/inhibition (E/I) balance in neurodevelopmental diseases. In vitro studies have suggested a differential binding ability of alpha- and beta-Nrxn1 isoforms with Nlgn proteins, often with contradictory results. Thus, it has been reported that the insertion of AS4 in beta-Nrxn1 fully abolishes binding with Nlgn1 containing the B site, the predominant form of Nlgn1, in pull-down experiments [22, 23] and cell-binding assays [24]. Moreover, expression of beta-Nrxn1 (-AS4) in non-neuronal cells induced glutamatergic differentiation, which was blocked by the insertion of AS4 in beta-Nrxn1 [24]. In contrast to the full inhibition imposed by AS4, other data obtained from similar experimental approaches have shown that the insertion of AS4 partly inhibits the binding of beta-Nrxn1 with Nlgn1 [22, 25, 26]. Furthermore, surface plasmon resonance (SPR) experiments have shown that beta-Nrxn isoforms can bind to Nlgn1-3 and that the insertion of AS4 decreases the binding affinity of beta-Nrxn1 for Nlgn1-3 [27]. Another study using a similar SPR approach showed that alpha-Nrxn1 isoforms containing or lacking AS4 did not bind to Nlgn1 containing the B site [22]. Whereas these findings have led to uncover an important role for the selection of alpha- or beta-Nrxn1 isoforms and splicing at AS4 in binding to Nlgns, these studies also face important limitations. For example, fusion proteins containing the soluble ectodomain of Nrxn proteins are commonly tested in binding assays with Nlgn-expressing cells, but Nrxns and Nlgns must interact as membrane proteins in opposing cells [28, 29]. Importantly, it has been shown that Nrxn binding, as tested in binding assays, is not sufficient for the synaptic function of Nlgn1, which requires transcellular oligomerization of Nrxn/Nlgn pairs [28]. Moreover, post-translational modifications in the form of heparan sulfate carbohydrates in the juxtamembrane region of Nrxn1 and the presence of extracellular ligands modulate the transcellular interactions of Nrxns with their postsynaptic receptors [30, 31, 32]. These cell-type specific modifications are not fully accomplished in heterologous assays performed in non-neuronal cells [32]. Finally, the preference of axonal alpha and beta-Nrxn1 isoforms for GABAergic Nlgn2 has been less extensively documented [33]. Therefore, a better understanding of *NRXN1* dysfunction in neurodevelopmental diseases would require analysis of the interactions displayed by Nrxn1 isoforms with postsynaptic partners at synaptic contacts as a step toward identifying the specific interactions affected in autism.

In this work, we analysed the presynaptic recruitment of alpha- and beta-Nrxn1 isoforms expressed in neurons by Nlgn1 and Nlgn2 located in an opposing membrane. We found that beta-Nrxn1 was recruited at presynaptic terminals induced by Nlgn1 and Nlgn2. In contrast, alpha-Nrxn1 isoforms were not recruited by Nlgn1 but, instead, recruited by Nlgn2 at presynaptic terminals. The insertion of AS4 decreased, but did not abolish, the recruitment of alpha and beta-Nrxn1 by Nlgn1 and Nlgn2. These data confirmed that beta-Nrxn1 is a presynaptic partner of Nlgn1 and Nlgn2, whereas alpha-Nrxn1 isoforms are partners of Nlgn2. To gain insight into the type of interactions affected in autism, we analysed the association of a dominant-negative mutant beta-Nrxn1 with Nlgn proteins in the brain of a mouse model of autism. Interestingly, we detected the formation of a protein complex involving mutant beta-Nrxn1 and Nlgn1 in the different forebrain areas analysed. Notably, Nlgn2 and Nlgn3 were not detected in the same protein complexes. These data indicated a selective pattern of interaction for a mutant beta-Nrxn1 with glutamatergic Nlgn1 in a mouse model of autism. Our data suggest that mutations in the *NRXN1* gene in autism could alter specific synaptic pathways through a mechanism dependent on the affected alpha- or beta-Nrxn1 isoform.

## Results

### Expression of epitope-tagged alpha and beta Nrxn1 isoforms in cultured neurons

**Fig. 1:**
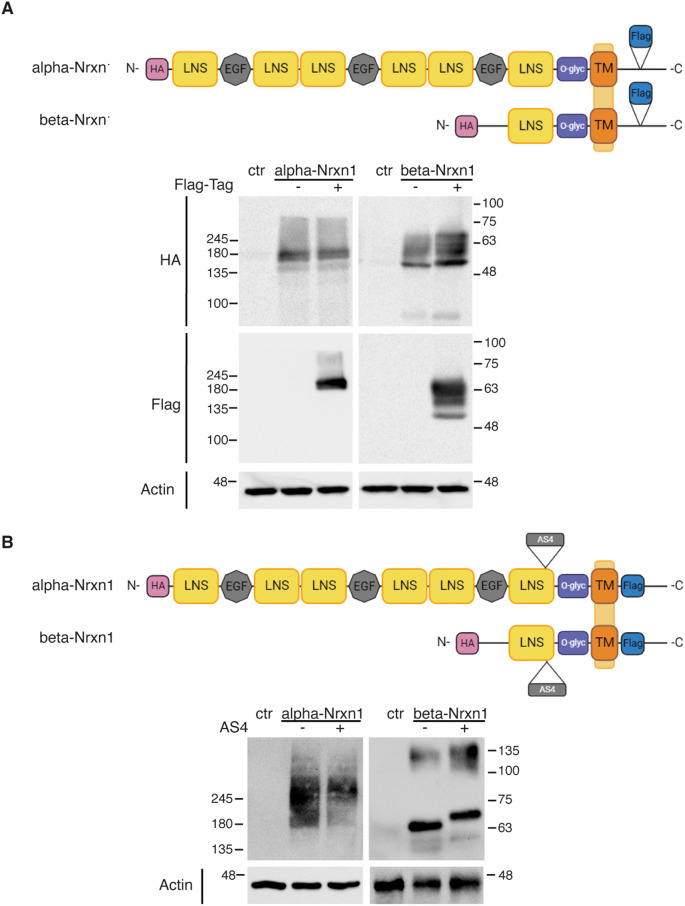
Generation of epitope-tagged alpha- and beta-Nrxn1 isoforms. (**A**) Comparison of the expression of Flag-tagged alpha- and beta-Nrxn1 with that of Flag-untagged versions. Schematic drawings showing the insertion site of a Flag-sequence in the cytoplasmic tail of alpha- and beta-Nrxn1 proteins. All the constructs contained an additional HA tag at the N-terminal region. Western blots of HEK293T cells transfected with Flag-tagged and untagged alpha and beta-Nrxn1 (-AS4) constructs were probed with Flag and HA antibodies, as indicated. (**B**) Expression of alpha- and beta-Nrxn1 splice isoforms. Drawings showing the generation of Flag-tagged alpha- and beta-Nrxn1 constructs containing (AS4) or lacking (-AS4) an insertion at splice site 4. Western blot experiments showing the expression of alpha- and beta-Nrxn1 splice isoforms in neuronal cultures transduced with lentiviral vectors specific for neuronal expression

The identification of the Nrxn1 isoforms that function as presynaptic receptors during synaptic differentiation has been limited by the absence of immunological tools for specific Nrxn1 splice isoforms and by the elevated number of Nrxn1 isoforms with high homology. To circumvent this problem, we inserted a short Flag sequence into the cytoplasmic tail of Nrxn1 (Fig. 1). Because the cytoplasmic domain is common to all Nrxn1 isoforms, we reasoned that this tagging approach would be better suited to establish comparisons among Nrxn1 proteins, which are highly polymorphic at the extracellular domains. The expression pattern of Flag-tagged alpha- and beta-Nrxn1 (-AS4) proteins in Western-blot experiments was similar to those of the same isoforms lacking the Flag-tag, indicating that the insertion of the Flag-sequence does not affect expression or transport (Fig. 1A). The molecular weight of Nrxn-1 bands was of 160–180 kDa for alpha-Nrxn1 (-AS4), and 55–70 kDa for beta-Nrxn1 (-AS4). The broad pattern of the bands and the slower mobility compared with the Nrxn-1 protein backbones (expected molecular weight: 163 kDa for alpha-Nrxn-1; 53 kDa for beta-Nrxn1) indicated some degree of glycosylation of Nrxn-1 proteins in HEK293T cells. Immunostaining experiments performed in non-permeabilized cells showed that the surface expression of Flag-tagged alpha and beta-Nrxn1 isoforms was indistinguishable from that of Nrxn1 isoforms lacking the tag, indicating that the insertion of the epitope tag does not affect surface expression (Suppl. Figure 1). Then, we generated lentiviral vectors to drive the neuronal expression of four Flag-tagged Nrxn1 isoforms: alpha-Nrxn-1 (AS4), alpha-Nrxn-1 (-AS4), beta-Nrxn-1 (AS4) and beta-Nrxn-1 (-AS4) (Fig. 1B). The human synapsin promoter included in these vectors drives specific expression in neurons, sparing expression in non-neuronal cells [34]. Therefore, this expression system ensures the specific post-translational modifications of Nrxn-1 proteins in neurons. In lysates of cortical neurons transduced with the lentiviral vectors, the anti-Flag antibody detected alpha-Nrxn1 proteins as broad bands of 180–250 kDa and beta-Nrxn1 as two main bands of approximately 70 kDa and 130–140 kDa (Fig. 1B). The higher molecular weight of alpha- and beta-Nrxn-1 proteins in cultured neurons when compared with the expression in HEK293T cells indicated a more complex glycosylation of Nrxn-1 proteins, which is consistent with the incorporation of heparan sulfate moieties in Nrxn-1, as previously reported [32]. Therefore, lentiviral-mediated neuronal-specific expression of epitope-tagged Nrxn1 isoforms allows to study of the presynaptic recruitment of specific Nrxn1 isoforms during synaptic differentiation.

### Recruitment of alpha-Nrxn1 isoforms at presynaptic terminals induced by Nlgn2

**Fig. 2:**
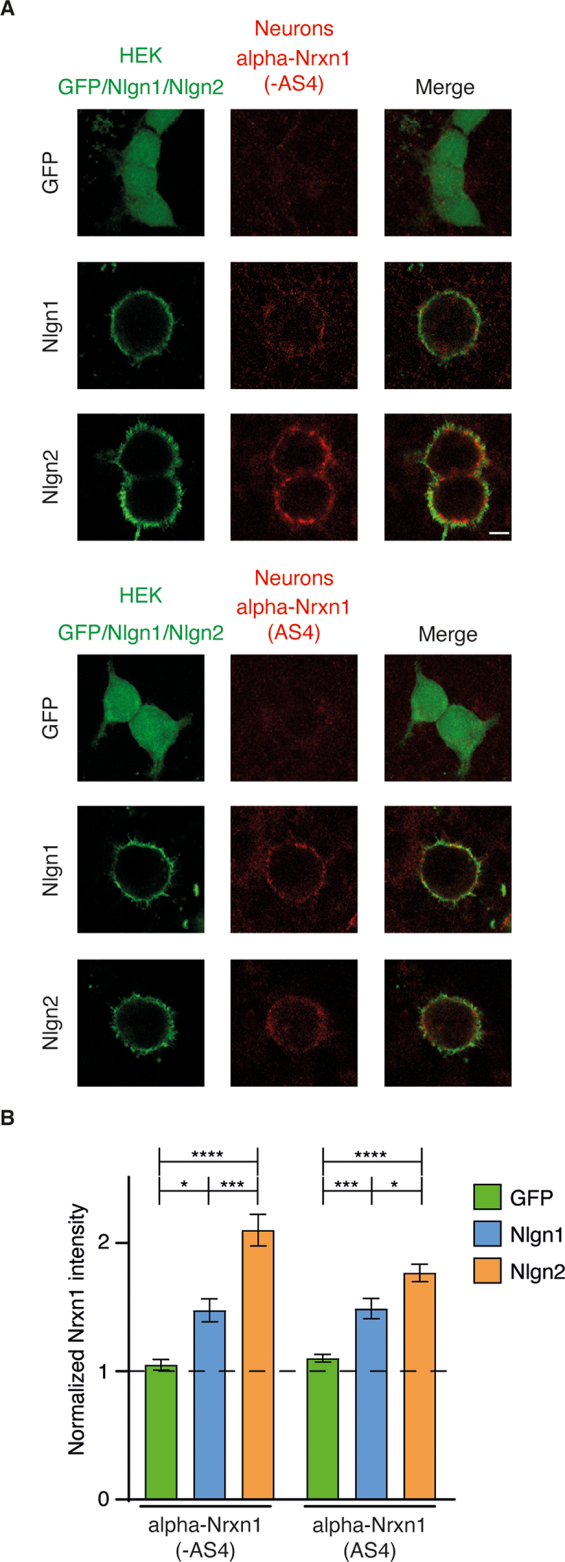
Preferential transcellular recruitment of axonal alpha-Nrxn1 by Nlgn2 in non-neuronal cells. **A**. Co-culture of cortical neurons expressing alpha-Nrxn1 (-AS4) or alpha-Nrxn1 (AS4) with HEK293T cells expressing GFP, Nlgn1 or Nlgn2, as indicated. Expression of alpha-Nrxn1 splice isoforms in neurons and Nlgn1/2 in non-neuronal cells was detected with Flag and VSV antibodies, respectively. **B**. Quantification of the alpha-Nrxn1 (-AS4) and alpha-Nrxn1 (AS4) signals recruited at contact sites induced by Nlgn1 and Nlgn2. Note that alpha-Nrxn1 isoforms are preferentially recruited by Nlgn2. The insertion of AS4 decreased the recruitment of alpha-Nrxn1 by Nlgn2. Scale bar, 5 μm for Nlgn1 and Nlgn2 and 6,85 μm for GFP transfections

The expression of Nlgn1 and Nlgn2 at postsynaptic terminals induces glutamatergic and GABAergic differentiation, respectively, through the recruitment of axonal Nrxns at contact sites [24, 28]. However, the identity of the Nrxn isoforms that are actively engaged at presynaptic terminals by postsynaptic Nlgns is not completely known. To analyse the presynaptic recruitment of alpha- and beta-Nrxn1 isoforms by Nlgn1 and Nlgn2 proteins, we adapted the co-culture assay originally developed by Scheiffele et al. [29]. For that purpose, cortical cultures were transduced with lentiviral vectors expressing Flag-tagged Nrxn1 isoforms and allowed to express the proteins for 7–9 days. Then, transduced neurons were co-cultured with non-neuronal cells expressing VSV-tagged Nlgn1 or Nlgn2 proteins. Owing to the high efficiency and neuronal specificity of lentiviral transduction, presynaptic recruitment of Nrxn1 proteins at Nlgn-mediated synaptic contacts can be analysed by immunostaining with Flag and VSV antibodies. We first studied the recruitment of alpha-Nrxn1 (-AS4) and alpha-Nrxn1 (AS4) by Nlgn proteins. Immunofluorescence experiments revealed lower recruitment of alpha-Nrxn1 (-AS4) and alpha-Nrxn1 (AS4) on Nlgn1-expressing cells compared with GFP-transfected cells (Fig. 2). However, the expression of Nlgn2 in non-neuronal cells induced a clear recruitment of alpha-Nrxn1 (-AS4) at the resulting presynaptic terminals, which was less intense for the alpha-Nrxn1 (AS4) isoform (Fig. 2). Quantification of the Nrxn1 signal on Nlgn-transfected cells confirmed the preferential recruitment of alpha-Nrxn1 by Nlgn2 and the partial inhibitory effect exerted by AS4 (normalized alpha-Nrxn1 signal on the transfected cells over non-transfected areas. Alpha-Nrxn1 (-AS4): GFP, 1.050 ± 0.0423; Nlgn1, 1.475 ± 0.0905; Nlgn2, 2.099 ± 0.1230. One-way ANOVA: F(2, 36) = 23.05, *p* < 0.0001. Post-hoc Tukey’s multiple comparisons test: Nlgn1 vs. GFP, *p* = 0.0438; Nlgn2 vs. GFP, *p* < 0.0001; Nlgn1 vs. Nlgn2, *p* = 0.0003. N 9–17. Alpha-Nrxn1 (AS4): GFP, 1.101 ± 0.0299; Nlgn1, 1.488 ± 0.07847; Nlgn2, 1.766 ± 0.06724. One-way ANOVA: F(2, 32) = 25.28, *p* < 0.0001. Post-hoc Tukey’s multiple comparisons test: Nlgn1 vs. GFP, *p* = 0.0005; Nlgn2 vs. GFP, *p* < 0.0001; Nlgn1 vs. Nlgn2, *p* = 0.0115. N 11–13) (Fig. 2). Therefore, alpha-Nrxn1 isoforms are preferentially recruited by Nlgn2 at presynaptic terminals.

### Presynaptic recruitment of beta-Nrxn1 isoforms by Nlgn1 and Nlgn2

**Fig. 3:**
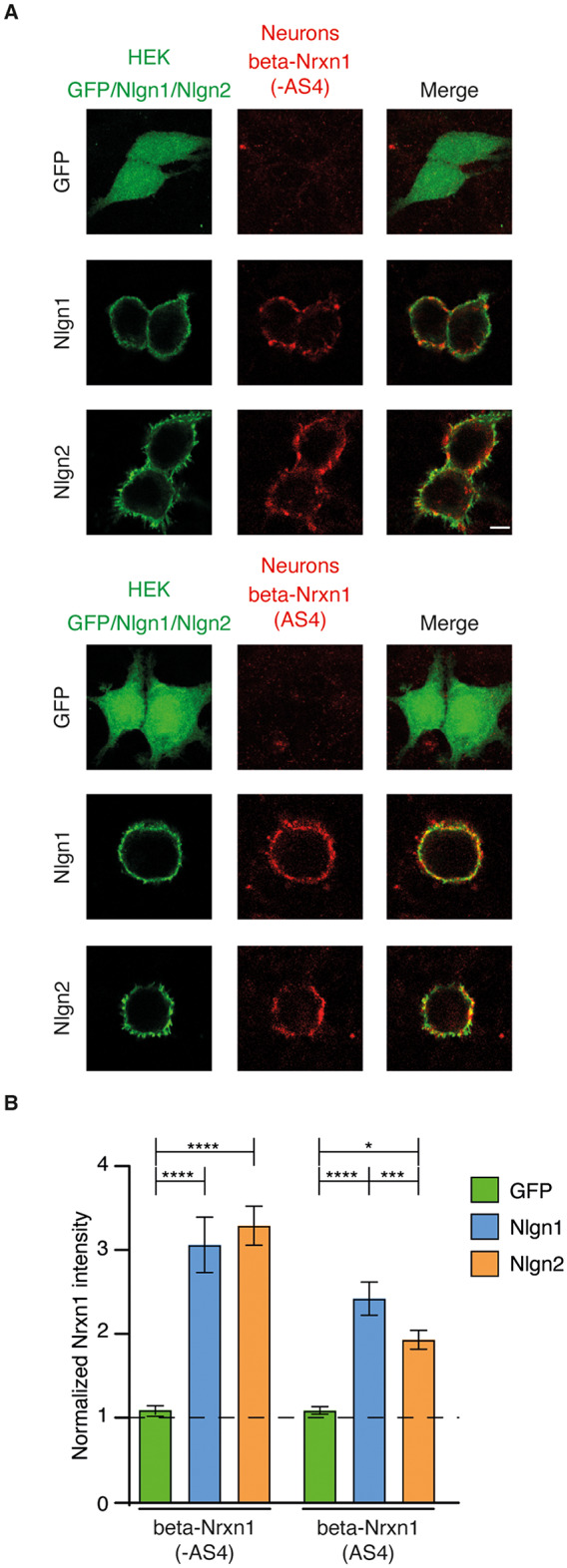
Transcellular recruitment of beta-Nrxn1 at presynaptic contacts mediated by Nlgn1 and Nlgn2. **A**. Cortical neurons expressing beta-Nrxn1 (-AS4) or beta-Nrxn1 (AS4) were co-cultured with HEK293T cells expressing GFP, Nlgn1 or Nlgn2, as shown. The expression of beta-Nrxn1 splice isoforms and Nlgn1/2 was detected with Flag and VSV antibodies, respectively. **B.** Graphs showing the quantification of neuronal beta-Nrxn1 splice isoforms recruited at presynaptic sites induced by Nlgn1 and Nlgn2. Note that beta-Nrxn1 proteins are similarly recruited by Nlgn1 and Nlgn2 and that the insertion of AS4 in beta-Nrxn1 decreases presynaptic recruitment. Scale bar, 5 μm for Nlgn1 and Nlgn2 and 6,85 μm for GFP transfections

To determine whether the recruitment of beta-Nrxn1 at presynaptic terminals obeys the same rules as for alpha-Nrxn1 isoforms, we transduced cortical neurons with beta-Nrxn1 (-AS4) and beta-Nrxn1 (AS4) followed by co-culture with non-neuronal cells expressing Nlgn1 or Nlgn2 proteins. In contrast to the results obtained for alpha-Nrxn1 isoforms, Nlgn1 promoted a robust presynaptic recruitment of beta-Nrxn1 (-AS4) (Fig. 3). The insertion of AS4 reduced the presynaptic recruitment of beta-Nrxn1 by Nlgn1 at contacting terminals (Fig. 3). We subsequently analysed the recruitment of beta-Nrxn1 isoforms by Nlgn2. We found that Nlgn2-expressing cells recruited presynaptic beta-Nrxn1 (-AS4) at similar levels as Nlgn1 (Fig. 3). Moreover, the insertion of AS4 decreased the presynaptic recruitment of beta-Nrxn1 by Nlgn2 (Fig. 3). Quantification of the data confirmed the similar recruitment of beta-Nrxn1 (-AS4) by Nlgn1 and Nlgn2 and the partial inhibition imposed by the insertion of AS4 (normalized beta-Nrxn1 signal on the transfected cells over non-transfected areas. Beta-Nrxn1 (-AS4): GFP, 1.091 ± 0.05129; Nlgn1, 3.062 ± 0.3296; Nlgn2, 3.292 ± 0.2319. One-way ANOVA: F(2, 40) = 20.63, *p* < 0.0001. Post-hoc Tukey’s multiple comparisons test. Nlgn1 vs. GFP, *p* < 0.0001; Nlgn2 vs. GFP, *p* < 0.0001; Nlgn1 vs. Nlgn2, *p* = 0.7783. N 11–19. Beta-Nrxn1 (AS4): GFP, 1.088 ± 0.04695; Nlgn1, 2.421 ± 0.1987; Nlgn2, 1.932 ± 0.1132. One-way ANOVA: F(2, 34) = 25.78, *p* < 0.0001. Post-hoc Tukey’s multiple comparisons test. Nlgn1 vs. GFP, *p* < 0.0001; Nlgn2 vs. GFP, *p* = 0.0001; Nlgn1 vs. Nlgn2, *p* = 0.0296) (Fig. 3). Therefore, beta-Nrxn1 isoforms are similarly recruited at presynaptic terminals by Nlgn1 and Nlgn2.

### Trans-synaptic recruitment of beta-Nrxn1, but not alpha-Nrxn1, by postsynaptic Nlgn1 in cortical neurons

**Fig. 4:**
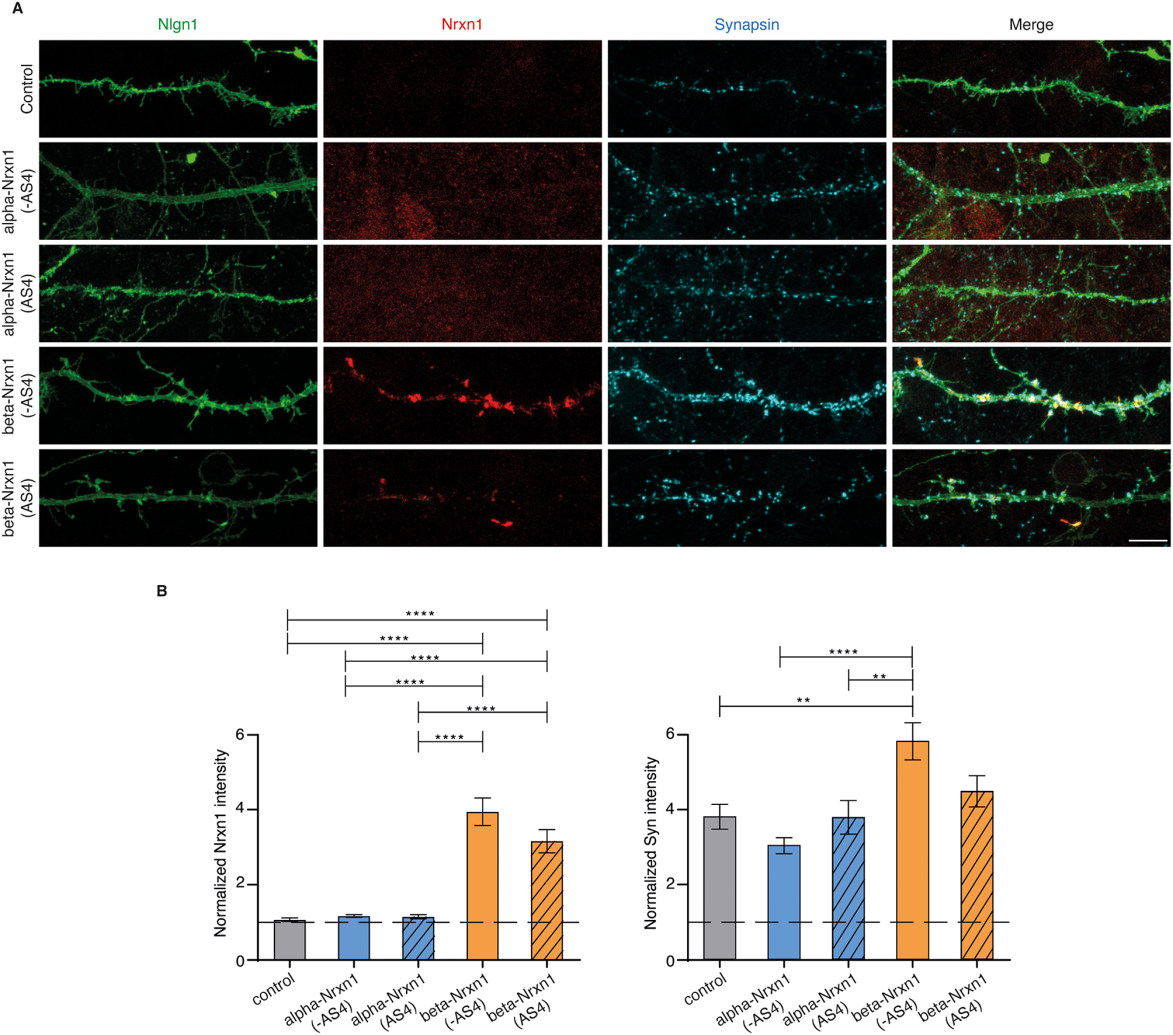
Nlgn1 expressed at postsynaptic terminals specifically recruits beta-Nrxn1, but not alpha-Nrxn1, at the presynapse. **A.** Panels showing the presynaptic recruitment of alpha- and beta-Nrxn1 splice isoforms induced by postsynaptic Nlgn1, as indicated. Nlgn1 was expressed in sparse neurons from cortical cultures expressing alpha- or beta-Nrxn1 isoforms by lentiviral transduction. As a control, Nlgn1 was expressed in non-transduced cultures. Expression of Nlgn1 and Nrxn1 isoforms was analysed with VSV and Flag antibodies, respectively. Synapses were identified with a synapsin antibody. **B**. Graphs showing the quantification of the Nrxn1 signal (left) and synapsin-positive area (right) at presynaptic terminals induced by postsynaptic Nlgn1. Scale bar, 10 μm

Previous data indicated that Nlgn1 preferentially recruits beta-Nrxn1 isoforms at glutamatergic terminals, whereas Nlgn2 can recruit alpha- and beta-Nrxn1 isoforms at GABAergic terminals. However, the trans-synaptic interaction of Nlgns with Nrxns can be positively or negatively modulated by factors that act on postsynaptic neurons, which could be missing in heterologous assays in non-neuronal cells [31, 35, 36]. To confirm the specific recruitment of beta-Nrxn1 isoforms by Nlgn1 in a synaptic context, we modified the experimental paradigm so that a pair of Nrxn/Nlgn candidate proteins can be positioned at the presynaptic and postsynaptic membranes, respectively (Fig. 4). With this aim, we transfected Nlgn1 into cortical cultures previously transduced with lentiviruses expressing Nrxn1 isoforms. This approach results in the expression of Nlgn1 in sparse neurons, which are contacted by axons expressing epitope-tagged Nrxn1 isoforms. Synapse density was analysed in the same cultures with a synapsin antibody. Interestingly, dendritic expression of Nlgn1 failed to recruit alpha-Nrxn1 (-AS4) or alpha-Nrxn1 (AS4) at the resulting presynaptic terminals, despite Nlgn1 greatly increased synapse density in the transfected neurons, as expected (Fig. 4). These data suggest that the glutamatergic differentiation induced by Nlgn1 is independent of alpha-Nrxn1 proteins. In contrast, postsynaptic Nlgn1 produced a robust concentration of beta-Nrxn1 (-AS4) and beta-Nrxn1 (AS4) at contacting presynaptic terminals (Fig. 4). Quantification of the data showed that the level of presynaptic recruitment of beta-Nrxn1 (-AS4) was higher than that of beta-Nrxn1 (AS4), further indicating a modulatory role of AS4 in the interaction with Nlgn1 (normalized Nrxn1 signal on Nlgn1-transfected dendrites over non-transfected areas. Control, 1.064 ± 0.05029; alpha-Nrxn1 (-AS4), 1.165 ± 0.03692; alpha-Nrxn1 (AS4), 1.145 ± 0.05500; beta-Nrxn1 (-AS4), 3.941 ± 0.3642; beta-Nrxn1 (AS4) 3.159 ± 0.3114. One-way ANOVA: F(4, 65) = 36.41, *p* < 0.000. Post-hoc Tukey’s multiple comparisons test. Alpha-Nrxn1 (-AS4) vs. alpha-Nrxn1 (AS4), *p* > 0.9999; alpha-Nrxn1 (-AS4) vs. beta-Nrxn1 (-AS4), *p* < 0.0001; alpha-Nrxn1 (-AS4) vs. beta-Nrxn1 (AS4), *p* < 0.0001; alpha-Nrxn1 (-AS4) vs. control, *p* = 0.9980; alpha-Nrxn1 (AS4) vs. beta-Nrxn1 (-AS4), *p* < 0.001; alpha-Nrxn1 (AS4) vs. beta-Nrxn1 (AS4), *p* < 0.0001; alpha-Nrxn1 (AS4) vs. control, *p* = 0.9991; beta-Nrxn1 (-AS4) vs. beta-Nrxn1 (AS4), *p* = 0.1198; beta-Nrxn1 (-AS4) vs. control, *p* < 0.0001; beta-Nrxn1 (AS4) vs. control, *p* < 0.0001). Moreover, the increase in synapse density produced by Nlgn1 was further potentiated in synapses expressing beta-Nrxn1 (-AS4) at presynaptic terminals (synapsin levels on the Nlgn1-transfected dendrites over non-transfected areas: Control, 3.772 ± 0.3320; alpha-Nrxn1 (-AS4), 3.008 ± 0.2109; alpha-Nrxn1 (AS4), 3.754 ± 0.4476; beta-Nrxn1 (-AS4), 5.764 ± 0.4928; beta-Nrxn1 (AS4) 4.44 ± 0.4113. One-way ANOVA F(4, 66) = 7.061. *p* < 0.0001. Post-hoc Tukey’s multiple comparisons test. Alpha-Nrxn1 (-AS4) vs. alpha-Nrxn1 (AS4), *p* = 0.7141; alpha-Nrxn1 (-AS4) vs. beta-Nrxn1 (-AS4), *p* < 0.0001; alpha-Nrxn1 (-AS4) vs. beta-Nrxn1 (AS4), *p* = 0.1191; alpha-Nrxn1 (-AS4) vs. control, *p* = 0.6553; alpha-Nrxn1 (AS4) vs. beta-Nrxn1 (-AS4), *p* = 0.0057; alpha-Nrxn1 (AS4) vs. beta-Nrxn1 (AS4), *p* = 0.7681; alpha-Nrxn1 (AS4) vs. control, *p* > 0.9999; beta-Nrxn1 (-AS4) vs. beta-Nrxn1 (AS4), *p* = 0.1424; beta-Nrxn1 (-AS4) vs. control, *p* = 0.0034; beta-Nrxn1 (AS4) vs. control, *p* = 0.7529) (Fig. 4). These data indicate a specific role of beta-Nrxn1 isoforms as functional presynaptic receptors during the glutamatergic differentiation induced by Nlgn1.

### Selective association of a mutant beta-Nrxn1 with Nlgn1 in a mouse model of autism

Autism-associated mutations in *NRXN1* might affect a synaptic function shared by all Nrxn1 isoforms or a subset of interactors, depending on the affected Nrxn1 isoform. However, information on the protein complexes formed by Nrxn1 proteins in the brain is still lacking. To obtain insights into the type of interactions affected by Nrxn1 in autism, we analysed the association of a beta-Nrxn1 mutant protein with Nlgns in a mouse model of autism. The beta-Nrxn1ΔC mouse is a validated animal model of autism that expresses a C-terminal truncated mutant beta-Nrxn1 (-AS4) in adult forebrain neurons [37]. The presence of the extracellular domain allows beta-Nrxn1ΔC protein to engage in trans-synaptic interactions at specific synapses, whereas the absence of the cytoplasmic tail inhibits neurotransmitter release, resulting in a dominant negative mutant for beta-Nrxn1 [37, 38]. Furthermore, beta-Nrxn1ΔC protein contains a short epitope-tag that allows for the isolation and characterization of the associated protein complexes by immunoprecipitation and Western-blot experiments.

**Fig. 5:**
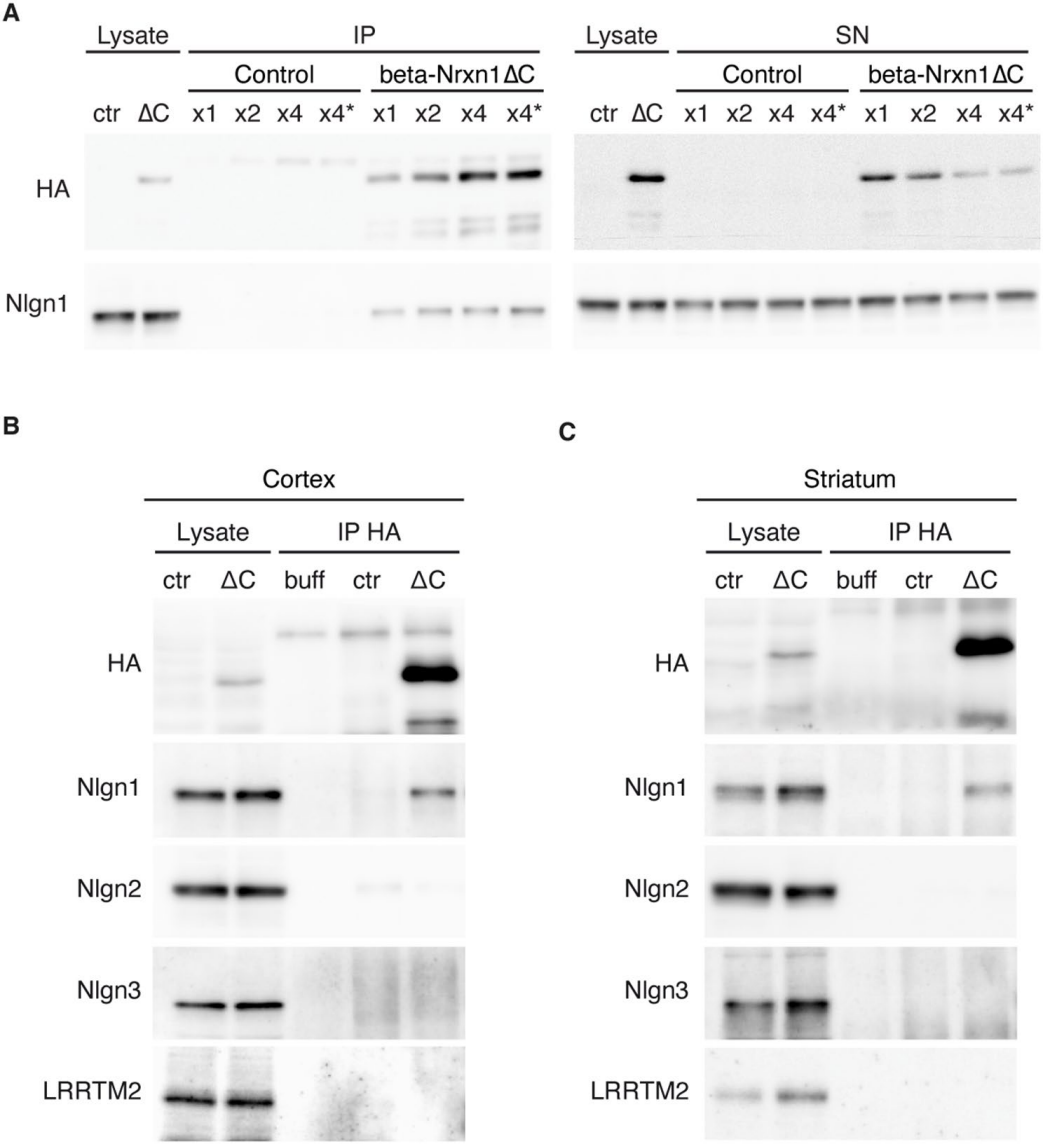
Selective association of a mutant beta-Nrxn1 protein with Nlgn1 in a mouse model of autism. **A**. Mutant beta-Nrxn1 was immunoprecipitated from the cortex of control and transgenic mice with increasing amounts of a HA antibody. The immunoprecipitates (IP) and the cleared supernatants (SN) were probed with HA and Nlgn1 antibodies, as shown. **B**,** C**. HA-immunoprecipitates from cortex (**B**) and striatum (**C**) of control and beta-Nrxn1ΔC adult mice were incubated with antibodies against HA, Nlgn1, Nlgn2, Nlgn3 and LRRTM2, as indicated. As a control, immunoprecipitation was performed in buffer alone (buff). Representative data from three (cortex) or two (striatum) independent experiments are shown. Averaged Nlgn1 levels in the co-immunoprecipitates as percentage of total Nlgn1 in the initial lysates: 5,01 ± 0,71 (cortex), and 1,74 ± 1,41 (striatum)

In a first set of experiments, we immunoprecipitated beta-Nrxn1ΔC protein from the mouse brain using increasing amounts of the specific antibody. Western-blot experiments revealed increased levels of immunoprecipitated beta-Nrxn1ΔC protein with increasing concentrations of the specific antibody. In parallel, beta-Nrxn1ΔC protein was cleared from the lysates, indicating efficient isolation (Fig. 5A). Interestingly, Nlgn1 was specifically detected in the beta-Nrxn1ΔC immunoprecipitates, and its levels increased in parallel with those of the recovered beta-Nrxn1ΔC protein (Fig. 5A). To analyse selectivity, we studied the presence of Nlgn1, Nlgn2 and Nlgn3 in the immunoprecipitates of beta-Nrxn1ΔC. Interestingly, Nlgn2 and Nlgn3 were not recovered in the cortical immunoprecipitates of the beta-Nrxn1ΔC mutant, although Nlgn1 was detected in the same immunoprecipitates (Fig. 5B). LRRTMs are glutamatergic postsynaptic proteins that interact with Nrxn1 (-AS4) isoforms [39, 40]. Notably, LRRTM2 was not detected in the immunoprecipitates of beta-Nrxn1ΔC, further suggesting a selective association with Nlgn1 at glutamatergic synapses (Fig. 5B). To analyse the association of beta-Nrxn1ΔC with Nlgn proteins in other brain regions, we performed additional immunoprecipitation experiments in the striatum, a region implicated in autism that shows expression of beta-Nrxn1ΔC in the mouse model [37]. Again, we found that Nlgn1 was co-immunoprecipitated with beta-Nrxn1ΔC from striatal lysates, whereas Nlgn2, Nlgn3 and LRRTM2 were not detected in the striatal immunoprecipitates (Fig. 5C). These data indicate the formation of a protein complex formed by a mutant beta-Nrxn1 and Nlgn1 in the brain of a mouse model of autism.

### Association of mutant beta-Nrxn1 with Nlgn1 initiates early after expression

**Fig. 6:**
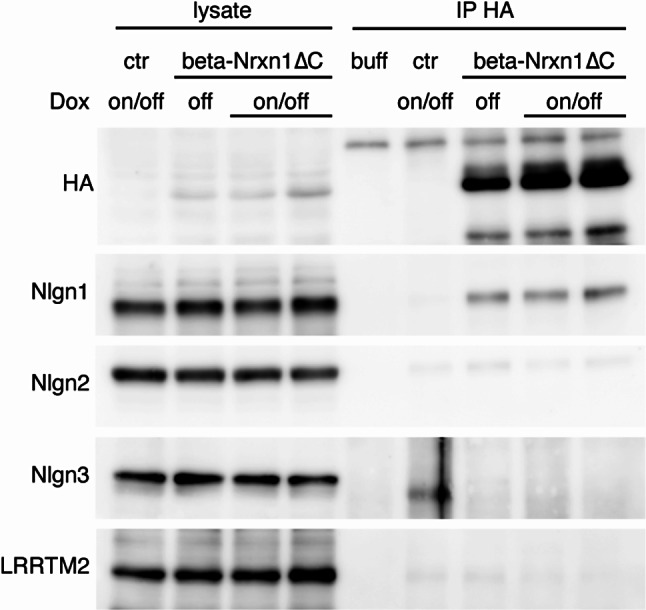
Association of mutant beta-Nrxn1ΔC with Nlgn1 initiates early after expression. HA-immunoprecipitates from the cortex of control and beta-Nrxn1ΔC mice left untreated (off DOX) or treated with DOX for 14 days and allowed to re-express the mutant beta-Nrxn1ΔC protein for 30 days (on/off DOX) were incubated with antibodies against HA, Nlgn1, Nlgn2, Nlgn3 and LRRTM2, as indicated. Nlgn1 is selectively recovered in beta-Nrxn1ΔC immunoprecipitates

The previous experiments were performed in adult mice following maintained expression of beta-Nrxn1ΔC protein starting at one month of age [37]. Therefore, we wondered whether the selective association of beta-Nrxn1ΔC with Nlgn1 results from a prolonged expression time. To test this possibility, we aimed to isolate beta-Nrxn1ΔC complexes from adult mice at an earlier time after beta-Nrxn1ΔC expression. The expression of beta-Nrxn1ΔC can be switched off in the adult brain with doxycycline (DOX) [37]. Thus, we turned off transgene expression with DOX for two weeks and then allowed beta-Nrxn1ΔC to be re-expressed in the absence of DOX for 30 days (on/off DOX; Fig. 6). This approach led to similar expression levels of beta-Nrxn1ΔC protein compared with the expression in non-treated transgenic mice (off DOX; Fig. 6). Interestingly, we similarly detected a specific association between beta-Nrxn1ΔC and Nlgn1 in the mouse brain (Fig. 6). Thus, Nlgn1 was specifically detected in the beta-Nrxn1ΔC cortical immunoprecipitates, whereas Nlgn2, Nlgn3 and LRRTM2 were not recovered in the same immunoprecipitates (Fig. 6). Together, these findings suggest a restricted pattern of association of a mutant beta-Nrxn1 with glutamatergic Nlgn1 in a mouse model of autism, which initiates early after expression and is maintained throughout adulthood.

## Discussion

In this study, we analysed the trans-synaptic interactions of alpha- and beta-Nrxn1 isoforms with Nlgn1 and Nlgn2 in cultured neurons and studied the type of association affected by mutant beta-Nrxn1 in a mouse model of autism. By using neuronal-specific expression of epitope-tagged Nrxn1 isoforms, combined with the expression of Nlgn1 and Nlgn2 in opposing membranes, we recapitulated the transcellular interaction of Nrxn/Nlgn at synapses. We found that glutamatergic differentiation induced by Nlgn1 specifically recruits beta-Nrxn1 at presynaptic terminals. In contrast, Nlgn2-mediated GABAergic differentiation induced the recruitment of alpha- and beta-Nrxn1 isoforms. The insertion of AS4 negatively modulates the presynaptic recruitment of Nrxn1 isoforms by Nlgn1 and Nlgn2. Importantly, the analysis of protein complexes associated with mutant beta-Nrxn1 suggested a selective dysfunction of the beta-Nrxn1/Nlgn1 pathway in a mouse model of autism. These findings suggest that dysfunction of beta-Nrxn1 in neurodevelopmental diseases could alter unique synaptic functions not shared by alpha-Nrxn1 isoforms.

Despite their major role at the synapse, most of what is known about the interactions of Nrxns with postsynaptic partners arises from in vitro experiments testing the binding of recombinant Nrxn proteins produced in non-neuronal cells with postsynaptic candidates [22, 24, 25, 26, 27]. However, it is well known that the interaction of Nrxns and Nlgns requires the formation of oligomers at the plasma membrane, a condition that is not accomplished in binding assays [28]. Therefore, we decided to develop an approach that ensures the axonal expression of Nrxn-1 proteins and, moreover, tests the interaction with Nlgn proteins across the cell surface. Compared with broadly used binding approaches, the experimental system used in this study offers several advantages. First, it assures the neuronal-specific post-translational mechanisms of Nrxn1 proteins, such as glycosylation, which is important for their function [32]. Second, the interaction of Nrxn1 isoforms with Nlgn proteins is studied in opposing membranes, reproducing the transcellular expression at the synapse [28]. Moreover, epitope-tagged Nrxn1 proteins are expressed by virtually all neurons in the culture, which facilitates the comparison of the same population of axons contacting Nlgn-expressing cells with non-contacting axons in the same field. Using this assay, we found that beta-Nrxn1 proteins are similarly recruited by Nlgn1 and Nlgn2 at presynaptic terminals. The insertion of AS4 in beta-Nrxn1 reduced, but did not inhibit, the recruitment of beta-Nrxn1 by Nlgn1 and Nlgn2. These data further support a modulatory role of the insertion of AS4 in the regulation of the interaction of beta-Nrxn1 with Nlgn1 and extend these observations to Nlgn2-mediated synaptic differentiation. In contrast, alpha-Nrxn1 proteins, either containing or lacking AS4, were not recruited at glutamatergic terminals induced by Nlgn1. Instead, alpha-Nrxn1 proteins were recruited by Nlgn2 during GABAergic differentiation. Again, the insertion of AS4 in alpha-Nrxn1 decreased the presynaptic recruitment mediated by Nlgn2. Owing to the differential functions of Nlgn1 and Nlgn2 at synapses, our data indicate a unique role of beta-Nrxn1 as presynaptic receptor for Nlgn1 at glutamatergic synapses and a shared role of alpha- and beta-Nrxn1 proteins as presynaptic partners for Nlgn2 during GABAergic differentiation. In accordance with a function of alpha-Nrxns at GABAergic synapses, a decrease in GABAergic synapse density has been shown in knockout mice lacking alpha-*Nrxn1/2/3* genes [41]. Our data are in agreement with a major role for the selection of alpha- or beta-Nrxn1 isoforms in the regulation of the interaction with Nlgn1/2 compared with the modulatory function for the splicing at AS4 site.

The identification of mutations in *NRXN1* associated with neurodevelopmental disorders has pointed at a hypofunction of Nrxn1 isoforms in brain diseases. *NRXN1* mutations can affect gene regions common to alpha- and beta-Nrxn1 or specific sequences of alpha- or beta-Nrxn1 isoforms. On the basis of these observations, the loss of Nrxn1 function in autism could alter a common function shared by all Nrxn1 isoforms or unique functions in an isoform-dependent manner. We have found experimental evidence for the latter possibility. The beta-Nrxn1ΔC mouse is an animal model of autism that expresses a C-terminal truncated mutant beta-Nrxn1 (-AS4) protein in adult forebrain neurons. Beta-Nrxn1ΔC mice exhibit increased repetitive behaviour, a lack of social interaction and an exaggerated response to non-social stimuli, resembling the main features of autism [37]. The presence of an intact extracellular domain allows beta-Nrxn1ΔC protein to associate with postsynaptic ligands, whereas the absence of the intracellular domain inhibits neurotransmitter release, resulting in a dominant negative mutant for beta-Nrxn1 at targeted synapses [37, 38]. Based on the interaction displayed by beta-Nrxn1 in the neuronal assays, mutant beta-Nrxn1ΔC could associate with Nlgn1, Nlgn2, both or neither. We detected a specific association between mutant beta-Nrxn1ΔC and Nlgn1 in the mouse brain. The beta-Nrxn1ΔC/Nlgn1 protein complex was detected in the cortex and striatum, it was present from an early time point after expression and was maintained into adulthood. Nlgn3, an Nlgn-family member present at glutamatergic and GABAergic synapses, did not associate with mutant beta-Nrxn1ΔC. Moreover, LRRTM2, a postsynaptic protein of glutamatergic synapses that binds in vitro to beta-Nrxn1 (-AS4) with similar affinity than Nlgn1 [40], was not detected in the same protein complexes. These findings indicate that, rather than affecting a full repertoire of postsynaptic partners, dysfunction of beta-Nrxn1 results in ligand selectivity for Nlgn1 in a mouse model of autism. Although the reasons for the preferred interaction of beta-Nrxn1ΔC with Nlgn1 in the mouse model remain to be known, several factors might contribute such as a higher affinity for Nlgn1 in vivo, Nlgn1 availability or the presence of modifying factors, among others. The impaired beta-Nrxn1/Nlgn1 signalling would be expected to reduce the response of glutamatergic synapses and may well explain the decrease in glutamatergic transmission reported in the cortex of beta-Nrxn1ΔC mice [37]. The fact that alpha-Nrxn1 isoforms do not participate in the glutamatergic differentiation mediated by Nlgn1 suggested an unshared mechanism for beta-Nrxn1 dysfunction in a mouse model of autism. Interestingly, point mutations abolishing the specific translation initiation sequence of beta-Nrxn1, which is not shared by alpha-Nrxn1, have been identified in patients with autism, suggesting a selective loss of beta-Nrxn1 function [11, 12]. These data suggest that disease-associated mutations affecting beta-Nrxn1 isoforms, either alone or in combination with a loss of alpha-Nrxn1 function, could affect the E/I balance through a mechanism not compensated by alpha-Nrxn1 (i.e., reducing excitation at targeted glutamatergic synapses). The correction of the E/I imbalance associated with *NRNX1* dysfunction in autism might probably require different approaches in an isoform-dependent manner.

## Methods

### Experimental animals

Animal studies were performed in accordance with Directive 2010/63/UE regarding the use of experimental animals (Real Decreto 53/2013) and under consideration of the ARRIVE guidelines. All procedures were approved by the Committee of Animal Use for Research at the University of Seville (Spain). Animals were housed in the facilities of the Animal Production and Experimentation Service at the Institute of Biomedicine of Seville (IBiS) (registered and authorized center ES410910008015) and kept on a 12 h dark/light cycle at 22ºC with food and water provided *ad libitum*. Where indicated, the mice were fed DOX in the diet (SAFE) for two weeks. Adult (6–7 months) male and female mice were used for the experiments.

### DNA and lentiviral vectors

The expression vectors used for VSV-tagged Nlgn1 (A, B) and Nlgn2 and for HA-tagged alpha (-AS4), beta-Nrxn1 (-AS4) and beta-Nrxn1 (AS4) were previously described [28, 42, 43]. The alpha-Nrxn1 (AS4) vector was generated by inserting the AS4 sequence from beta-Nrxn1 (AS4) into alpha-Nrxn1 (-AS4). The octopeptide coding for the Flag-tag sequence was inserted between N1350 and G1351 of Nrxn1 isoforms (numbering corresponding to accession number NM_021767.2) by overlap-extension PCR using the following primers: primer 1, TATGCCATGTACAAGTACAGAAACC; primer 2, CGTCATCCTTGTAATCATTGGACTGTGCTGAGTTAC; primer 3, GGATGACGACGATAAGGGGGCTGTGGTCAAGGAG; and primer 4, CCCGGGGGTACCTCGAGT. The 16 nucleotides at the 5’ end of primers 2 and 3 code for the N-term and C-term two-thirds of the Flag-tag sequence, respectively, the first 8 nucleotides of which are complementary in primers 2 and 3. PCR 1 was performed with primer 1 and primer 2, and PCR 2 with primer 3 and primer 4 using an expression vector for Nrxn1 as a template. The amplified bands of PCR reactions 1 and 2 were purified and used in a PCR reaction that yielded a DNA fragment coding for the C-terminal region of Nrxn1 with the Flag-tag sequence inserted at N1350. This PCR fragment was cloned into expression vectors for alpha-Nrxn1 (AS4), alpha-Nrxn1 (-AS4), beta-Nrxn1 (AS4), and beta-Nrxn1 (-AS4) with restriction enzymes. The coding sequences of the four Flag-tagged Nrxn1 isoforms were subsequently cloned into a lentiviral vector under the control of a synapsin promoter [34]. The generation and concentration of recombinant lentiviral particles were performed as previously described [34]. The infection of cultured neurons with lentiviral particles was adjusted to achieve comparable expression levels for the Nrxn1 isoforms.

### Cell culture and immunofluorescence

Cortical cultures were obtained from the brain of E18-E19 rats of both sexes. Dissociated cells were plated in 24-well plates at 60,000 cells/well containing poly-D-lysine (Sigma Aldrich) coated glass coverslips and maintained in neurobasal medium (Invitrogen) supplemented with B27 (Thermo Fisher Scientific), GlutaMAX and penicillin/streptomycin (Invitrogen). HEK293T cultures were grown in Dulbecco’s modified Eagle medium (DMEM) containing 10% fetal bovine serum (HyClone), GlutaMAX and penicillin/streptomycin. Cortical cultures and HEK293T cells were transfected with Lipofectamine 2000 (Invitrogen) following the manufacturer’s recommendations. For cell surface staining, transfected HEK293T cells with Nrxn1 isoforms were fixed with 4% paraformaldehyde, incubated with anti HA antibodies, followed by permeabilization with 0.05% Triton X-100 and incubation with anti-Flag antibodies. For co-culture experiments, HEK293T cells transfected with VSV-Nlgn1, VSV-Nlgn2 or GFP vectors were trypsinized, counted and plated at 40–50,000 cells/well into 24-well plates containing cortical neurons. Co-cultures were maintained for 24 h, fixed with 4% paraformaldehyde and permeabilized with 0.05% Triton X-100. The following primary antibodies were used in immunofluorescence experiments: mouse anti-Flag M2 (Sigma Aldrich), rabbit anti-VSV (Sigma Aldrich) and guinea pig anti-synapsin (Synaptic System). As secondary antibodies, preabsorbed donkey antibodies conjugated with Cy2, Cy3 and Cy5 fluorophores (Jackson ImmunoResearch) were used. Images were acquired on a Leica Stellaris confocal microscope. Maximal projections of the Z-stacked images were analysed with ImageJ software. A mask covering the region of interest was created by drawing a line around the transfected cell (HEK293T or dendrite). The mask was duplicated in neighboring areas of the same field containing no transfected cells and was used as a control. Specific markers were quantified as the mean intensity on the transfected cell normalized to control intensity in the vicinity above a threshold value. Thresholds were set such that most of the specific signal was included. Statistical analyses were performed with GraphPad Prism software. Differences in means across multiple groups were analysed using one-way ANOVA followed by the *post hoc* Tukey’s test. P-values less than 0.05 were considered statistically significant. All data are presented as the mean ± SEM.

### Biochemical analysis

Cell cultures and forebrain tissues from the cortex or striatum were homogenized in lysis buffer (20 mM Tris-HCl pH 7.5; 100 mM NaCl; 5 mM MgCl_2_; 5 mM CaCl_2_ and 1% Triton X-100) containing a protease inhibitor cocktail (Roche). For immunoprecipitation experiments, forebrain lysates were first cleared with Protein-G Sepharose (GE Healthcare), followed by incubation with an HA antibody (Roche, clone 3F10). The immune complexes were isolated with Protein-G Sepharose, eluted in elution buffer (180 mM Tris-HCl pH 6.8 and 2% SDS) and resolved in SDS-PAGE gels. In each experiment, the loaded lysates corresponded to 8% or 16% of the total lysate volume used for immunoprecipitation. Western-blot experiments were performed using the following primary antibodies: mouse anti C-terminal Nlgn1 (NeuroMab, clone N97A/31); mouse anti Nlgn2 (Synaptic Systems, clone 5E6); mouse anti Nlgn3 (NeuroMab, clone N110/29); mouse anti LRRTM2 (NeuroMab, clone N209C/35); and rat anti-HA (clone 3F10, Roche, Basel, Switzerland). Immunoreactivity was detected with appropriate secondary antibodies conjugated with horseradish peroxidase (Jackson ImmunoResearch). Chemiluminescence was developed using Clarity ECL Substrate (Bio-Rad) or Clarity Max ECL Substrate (Bio-Rad) on a ChemiDoc Touch Imaging System (Bio-Rad).

## Electronic supplementary material

Below is the link to the electronic supplementary material.

Supplementary Material 1: Suppl. Figure 1 Surface expression of Flag-tagged Nrxn1 constructs. Description of data: Images show HEK293T cells transfected with alpha-Nrxn1 (-AS4) and beta-Nrxn1 (-AS4) either containing or lacking a Flag tag at the cytoplasmic domain, as indicated. Cells were surface-stained with anti HA antibodies in non-permeabilizing conditions, followed by permeabilization and incubation with an anti-Flag antibody. All constructs contain an HA epitope at the N-terminus of the extracellular domain. Surface staining was not affected by the insertion of a Flag tag in Nrxn1. Nuclei were stained with DAPI. Scale bar, 5 μm.

## Data Availability

No datasets were generated or analysed during the current study.

## Abbreviations

AS4: Alternative spliced segment 4, neurexin genes
DOX: Doxycycline
EGF: Epidermal growth factor-like domain
E/I: Excitation/inhibition balance.
LNS: Laminin, Neurexin, Sex-hormone binding globulin domain
Nlgns: Neuroligin proteins
*NLGN1*, *2*, *3*, *4X*, *4Y*: Human neuroligin genes 1, 2, 3, 4X and 4Y
Nrxns: Neurexin proteins
*NRXN1*-*3*: Human neurexin genes 1, 2 and 3
SPR: Surface plasmon resonance
